# Supplementary material for: Causal interplay between lactose intolerance and gut microbiota: a combined bidirectional Mendelian randomization and in vivo validation study
Source: Front Nutr. 2026 Jun 1;13:1803337. doi: 10.3389/fnut.2026.1803337 (PMC13265576; doi:10.3389/fnut.2026.1803337)
Supplement: Supplementary file 1 [file Data_Sheet_1.zip › supplementary materials/Forward/leave one out cross validation/ebi-a-GCST90027561.finngen_R12_E4_LACTONAS.pdf]

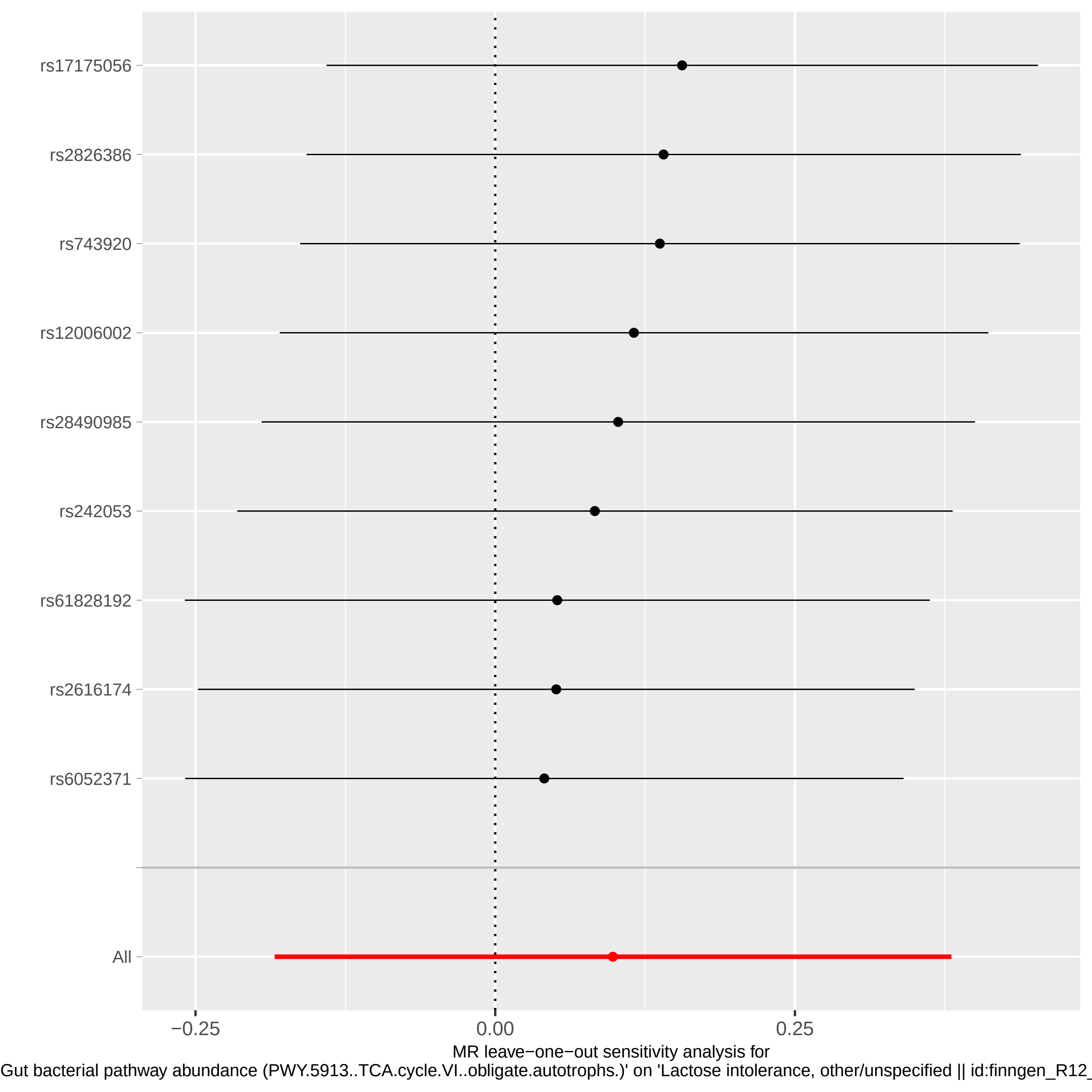

rs17175056
rs2826386
rs743920
rs12006002
rs28490985
rs242053
rs61828192
rs2616174
rs6052371
All
−0.25
0.00
0.25
MR leave−one−out sensitivity analysis for
'Gut bacterial pathway abundance (PWY.5913..TCA.cycle.VI..obligate.autotrophs.)' on 'Lactose intolerance, other/unspecified || id:finngen_R12_
